# Supplementary material for: Evaluation of DNA barcoding reference databases for marine species in the western and central Pacific Ocean
Source: PeerJ. 2025 Jul 14;13:e19674. doi: 10.7717/peerj.19674 (PMC12269782; doi:10.7717/peerj.19674)
Supplement: Supplemental Information 3 [file peerj-13-19674-s003.pdf]

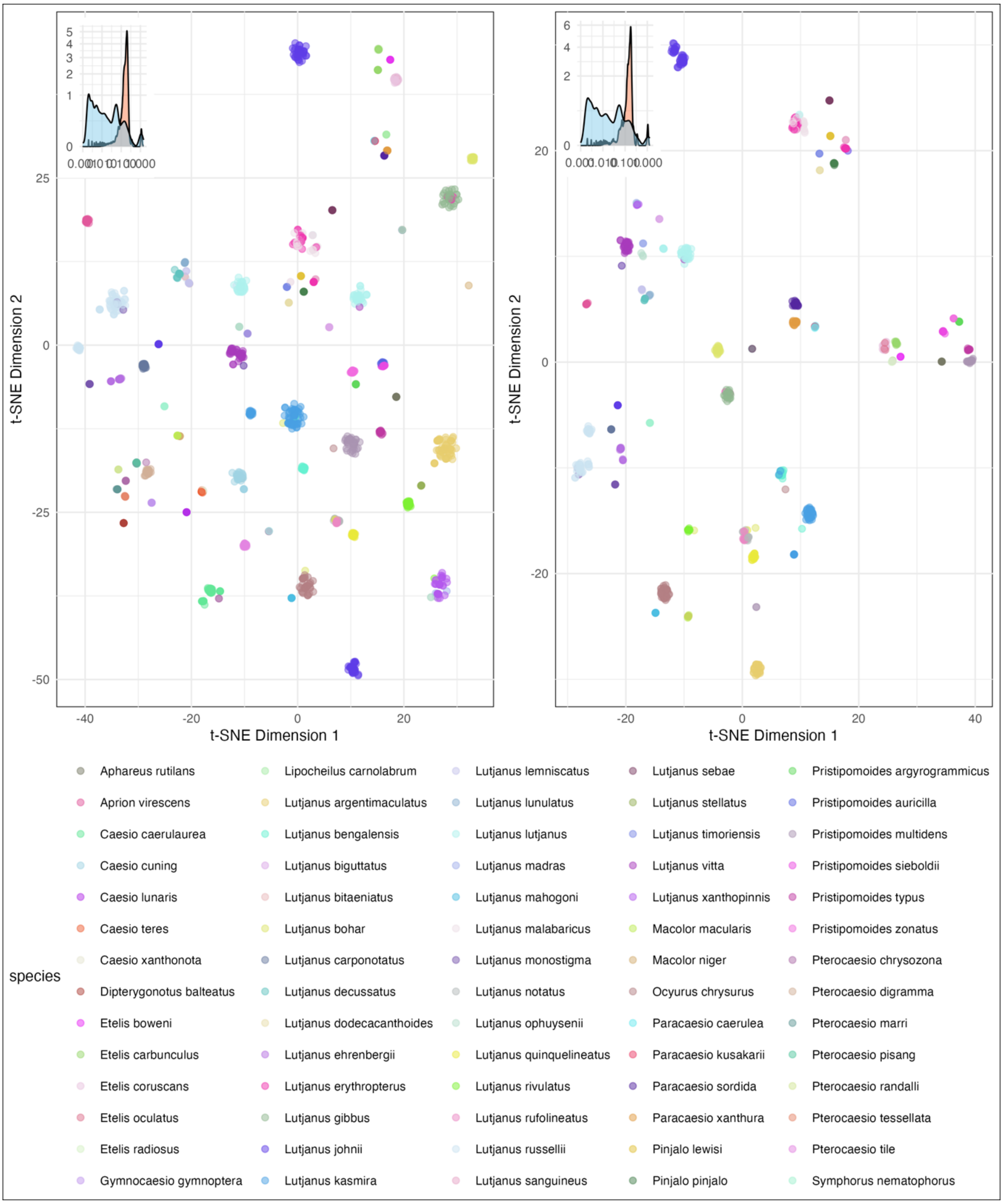

Supplementary figure 1. t-SNE clustering of sequences from NCBI (left) and BOLD (right) for Lutjanidae species.

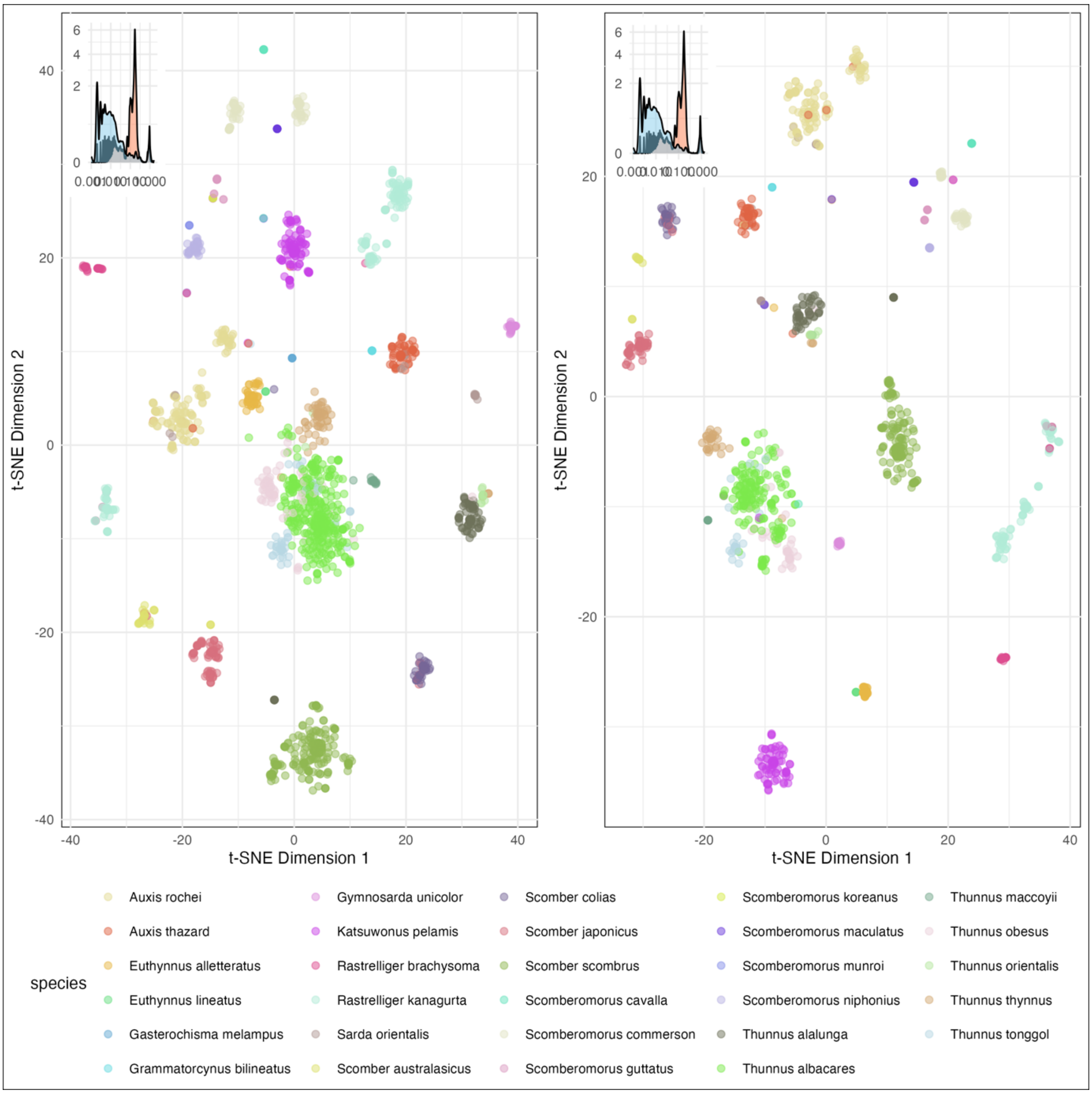

Supplementary figure 2. t-SNE clustering of sequences from NCBI (left) and BOLD (right) for Scombridae species.
